# Paediatric focal intracranial suppurative infection: a UK single-centre retrospective cohort study

**DOI:** 10.1186/s12887-019-1486-7

**Published:** 2019-04-25

**Authors:** Fabian J. S. van der Velden, Alexandra Battersby, Lucia Pareja-Cebrian, Nicholas Ross, Stephen L. Ball, Marieke Emonts

**Affiliations:** 1 Paediatric Immunology, Infectious Diseases and Allergy Department, Newcastle upon Tyne Hospitals NHS Foundation Trust, Great North Children’s Hospital, Newcastle upon Tyne, NE1 4LP UK; 2 Erasmus MC, Rotterdam, 3015 CE The Netherlands; 3 Microbiology Department, Newcastle upon Tyne Hospitals NHS Foundation Trust, Royal Victoria Infirmary, Newcastle upon Tyne, NE1 4LP UK; 4 Neurosurgery department, Newcastle upon Tyne Hospitals NHS Foundation Trust, Royal Victoria Infirmary, Newcastle upon Tyne, NE1 4LP UK; 5 Otorhinolaryngology, Newcastle upon Tyne Hospitals NHS Foundation Trust, Royal Victoria Infirmary, Newcastle upon Tyne, NE1 4LP UK; 6 Institute of Cellular Medicine, Newcastle University, Newcastle upon Tyne, NE2 4HH UK

**Keywords:** Paediatrics, Brain abscess, Empyema, subdural, Antimicrobials

## Abstract

**Background:**

Paediatric focal intracranial suppurative infections are uncommon but cause significant mortality and morbidity. There are no uniform guidelines regarding antibiotic treatment. This study reviewed management in a tertiary healthcare centre in the United Kingdom and considers suggestions for empirical treatment.

**Methods:**

A retrospective, single-centre cohort review of 95 children (< 18 years of age) with focal intracranial suppurative infection admitted between January 2001 and June 2016 in Newcastle upon Tyne, United Kingdom**.** Microbiological profiles and empirical antibiotic regimens were analysed for coverage, administration and duration of use. Mortality and neurological morbidity were reviewed. Data was analysed using t-tests, Mann-Whitney U tests, independent-samples median tests, and χ^2^-tests where appropriate. *P*-values < 0.05 were considered statistically significant.

**Results:**

Estimated annual incidence was 8.79 per million. Age was bimodally distributed. Predisposing factors were identified in 90.5%, most commonly sinusitis (42.1%) and meningitis (23.2%). Sinusitis was associated with older children (*p* < 0.001) and meningitis with younger children (p < 0.001). The classic triad was present in 14.0%.

43.8% of 114 isolates were *Streptococcus spp.*, most commonly *Streptococcus milleri* group organisms. Twelve patients cultured anaerobes.

Thirty one empirical antibiotic regimens were used, most often a third-generation cephalosporin plus metronidazole and amoxicillin (32.2%). 90.5% would have sufficient cover with a third generation cephalosporin plus metronidazole. 66.3% converted to oral antibiotics. Median total antibiotic treatment duration was 90 days (interquartile range, 60–115.50 days).

Mortality was 3.2, 38.5% had short-term and 24.2% long-term neurological sequelae.

**Conclusions:**

Paediatric focal intracranial suppurative infection has a higher regional incidence than predicted from national estimates and still causes significant mortality and morbidity. We recommend a third-generation cephalosporin plus metronidazole as first-choice empirical treatment. In infants with negative anaerobic cultures metronidazole may be discontinued.

## Introduction

Focal intracranial suppurative infections are serious conditions rarely seen in children. [1–5] They are divided in three categories: brain abscess (BA), subdural empyema (SDE), and extradural empyema (EDE). In the pre-antibiotic era, mortality reached nearly 100% [6], decreasing to around 36–60% in the 1970s when antibiotics became more readily available. [3, 7] Since then mortality has dropped further, ranging 3.7–24%, as computed tomography was introduced and cranial imaging improved, and metronidazole became part of most standard empirical regimens in the 1980s. [7–14]

Although mortality has dropped, these infections continue to cause significant mortality and morbidity, and can lead to rapid patient deterioration, thus adequate diagnostics and early aggressive treatment are crucial to optimise chances of complete recovery. [4, 9, 10, 14–16]

The annual estimated incidence is 4–5.3/1,000,000 [2, 12], with higher incidences in developing countries. [15, 17] For the United Kingdom (UK) this translates to approximately 3 paediatric cases per tertiary healthcare centre annually.

In 74.4–91.6% a predisposing factor can be determined. [7, 14, 18] Compared to adults, congenital heart disease (CHD) and immunosuppression occur more frequently in children. [2] Other common predispositions in children are meningitis and sinusitis. [4, 9, 16, 17] Patients can present with a variety of symptoms. The classic triad consisting of headache, fever, and focal neurological deficits is reported in 8.4–20% of children. [3, 12, 14, 18] Treatment is multidisciplinary consisting of antibiotics and neurosurgery. [3, 5] Neurosurgical intervention aids pathogen identification, reduces lesion size and decompresses, aiming to reduce effects on surrounding structures. [19]

Several microorganisms have been implicated, but most commonly cultured are *Streptococci* and *Staphylococci*. [1, 2, 10, 12, 14, 16, 20–22] 11.1–33% of patients grow anaerobic species [3, 7, 12], and 14.3–27% of BAs is polymicrobial. [11, 14] Empirical antibiotic treatment is broad-spectrum to cover these organisms, and rationalised on microbiological guidance.

There is no standardised empirical antibiotic treatment. [9, 16, 23, 24] Often first-choice empirical treatment is a third-generation cephalosporin plus metronidazole. [12, 19] These antibiotics have adequate pharmacokinetics and pharmacodynamics to achieve therapeutic concentrations within the central nervous system. [25]

In the UK there are currently no national guidelines regarding treatment of paediatric suppurative intracranial infections. [12] Internationally proposed consensus documents and guidelines are controversial [19, 23, 24, 26], because they are based on combined adult and paediatric literature. Paediatric evidence remains sparse, mainly consisting of case-series with small patient populations. [3, 11, 18, 21]

Published guidelines suggest antibiotic treatment for 6–8 weeks by intravenous administration only. [24] However, guidelines from The Infection in Neurosurgery Working Party of the British Society for Antimicrobial Chemotherapy [27] recommend 1–2 weeks of intravenous administration and to consider conversion to oral administration, depending on clinical response and decreasing C-reactive protein (CRP), to complete the antibiotic course.

This study evaluated local management of paediatric focal intracranial suppuration and reviewed antibiotic practice in order to consider empirical antibiotic guidelines.

## Methods

This single-centre retrospective cohort study reviewed paediatric patients with BA, SDE, and EDE, admitted to the Great North Children’s Hospital (GNCH), a tertiary healthcare centre for paediatric infectious diseases and neurosurgery in the North East of England between January 2001 and June 2016. Local Caldicott approval was obtained.

### Patient identification

Eligible cases were identified by assessing the paediatric infectious diseases, intensive care (PICU) and neurosurgery records, the previously described local cohort [9], and the hospital clinical coding database.

The following World Health Organization International Classification of Disease Codes [28], tenth revision, were used: G06.0 Intracranial abscess and granuloma, G06.2 Extradural and subdural abscess, unspecified, and G07 Intracranial and intraspinal abscess and granuloma in diseases classified elsewhere.

Cases were included if the patient was < 18 years of age on admission, had a confirmed diagnosis according to clinical information, and had diagnosis confirmation by radiology.

### Data collection

Data was collected from medical records and electronic systems used within the hospital. Data was recorded on patient demographics, admission duration, confirmed diagnosis, presenting symptoms, symptom duration before admission, predisposing factors, laboratory and microbiology results, antibiotics, neurosurgical management, morbidity and mortality.

#### Microbiology

Microbiological data was collected from cultures taken during admission. This included blood, cerebrospinal fluid (CSF), intracranial pus and paranasal sinus cultures.

Positive cultures were defined as cultures with microorganism growth or positive polymerase chain-reaction (PCR) (PCR panel: Streptococcus spp., Neisseria meningitides, Fusobacterium spp., Aspergillus spp. Staphylococcus spp). Antibiotic resistance patterns were recorded.

Cases without growth in any cultures, had cultures reassessed. As per microbiology protocol, gram stains of every sample are examined microscopically before culturing and sent for PCR identification if no growth is observed after incubation. If either test was found positive, the culture was considered positive. Otherwise, the culture was considered negative. Due to changes in the microbiology protocol between 2001 and 2016, not all samples were sent for PCR in growth-negative cases owing to increasing availability of PCR since 2004 only.

### Statistical analysis

Data was analysed with SPSS®Statistics, version 22 (IBM Corporation, Armonk, New York). Normality was assessed with the Shapiro-Wilk test. Normally distributed data was analysed with unpaired t-tests or one-sample t-tests. Not normally distributed data was analysed with Mann-Whitney U tests and independent-samples median tests. χ^2^-tests were used where appropriate. *P*-values < 0.05 were considered statistically significant.

## Results

**Fig. 1:**
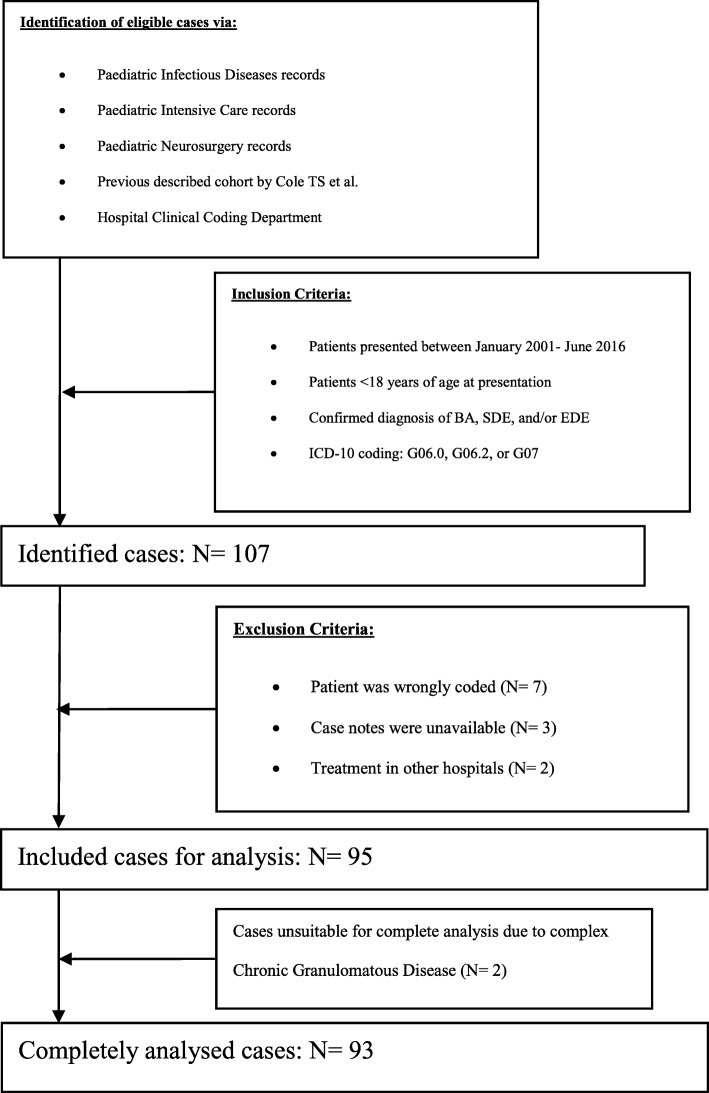
Flowchart of Patient Identification

107 eligible cases were identified. 7 cases were excluded due to incorrect coding, case notes were unavailable for 3, and 2 patients were primarily treated in other healthcare centres. 95 cases were suitable for analysis, of which 2 had chronic granulomatous disease (CGD) whose data were only suitable for diagnosis, predisposing factors and mortality analysis (Fig. 1).

This centre treated on average 6.13 cases annually. All patients originated from the GNCH catchment area. The child population (aged 0–19 years) of this area was 697,200 in 2014. [29, 30] This leads to an estimated annual incidence of 8.79/1,000,000.

### Patient demographics

**Fig. 2:**
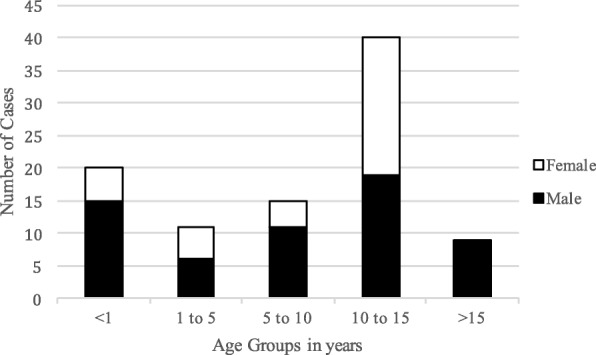
Case Distribution in Age Groups

There were 60 males (63.2%). Median admission age was 10.21 years (interquartile range (IQR), 1.57–12.67 years) and was bimodally distributed (Figure *2*). Age at presentation was not significantly different between males and females (median, 8.73 and 11.53 years, respectively, *p* = 0.172). There was no association between age or sex and type of intracranial suppurative infection.

### Diagnosis and localisation

All patients underwent cranial imaging with contrast. 33/95 patients had BA (34.7%), 46/95 had SDE (48.4%), 2/95 had EDE (2.2%), 6/95 had BA and SDE (6.3%), 1/95 had BA and EDE (1.1%), and 7/95 had SDE and EDE (7.4%). 13/40 (32.5%) had multiple abscesses. The majority of brain abscesses was located in the frontal lobe (50%).

### Predisposing factors

**Fig. 3:**
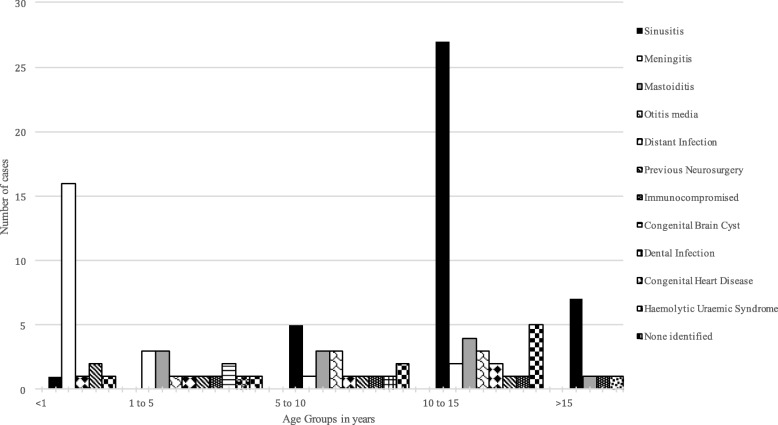
Predisposing Factors by Age Group

Predisposing factors were identified in 86 patients (90.5%). Most common were sinusitis (42.1%) and meningitis (23.2%) (Table 1). 27/40 sinusitis patients were in the 10–15 years age group and 16/22 meningitis patients were in < 1-year age group (Fig. 3). Meningitis patients were younger compared to children with other predisposing factors (median, 0.46 versus 11.55 years, *p* < 0.001) and sinusitis patients were older compared to children with other predisposing factors (median, 12.47 versus 2.64 years, p < 0.001). Other predisposing factors were not associated with age.

**Table 1:** Predisposing Factors (*N* = 95)

Predisposing Factor	N (%)	Predisposing Factor	N (%)
Sinusitis	40 (42.1%)	Immunocompromised	4 (4.2%)
Meningitis	22 (23.2%)	Congenital Brain Cyst	2 (2.1%)
Mastoiditis	11 (11.6%)	Congenital Heart Disease	1 (1.1%)
Otitis media	7 (7.4%)	Dental Infection	1 (1.1%)
Previous neurosurgery	5 (5.3%)	Haemolytic Uraemic Syndrome	1 (1.1%)
Distant Infection	5 (5.3%)	None Identified	9 (9.5%)

### Presenting symptoms

For 89 patients symptom duration was documented and the median duration was 7 days (IQR 5–14 days). Most common symptoms were history of fever (66.7%), vomiting (66.7%), and headache (58.1%) (Table 2). The classical triad combining these symptoms was present in 13 patients (14.0%).

**Table 2:** Presenting Symptoms (*N* = 93)

Symptom	N (%)	Symptom	N (%)
Fever (History)	62 (66.7%)	Nuchal Rigidity	14 (15.1%)
Vomiting	62 (66.7%)	Rhinorrhoea	13 (14.0%)
Headache	54 (58.1%)	Papilloedema	11 (11.8%)
Focal Neurological Deficit	35 (37.6%)	Photophobia	10 (10.8%)
Fever (> 38.0 **°**C)	33 (35.5%)	Nausea	7 (7.5%)
Lethargy	33 (35.5%)	Increased Head Circumference	5 (5.4%)
Altered Level of Consciousness	27 (29.0%)	Behavioural Change	5 (5.4%)
Seizure	22 (23.7%)		

### Microbiology

**Fig. 4:**
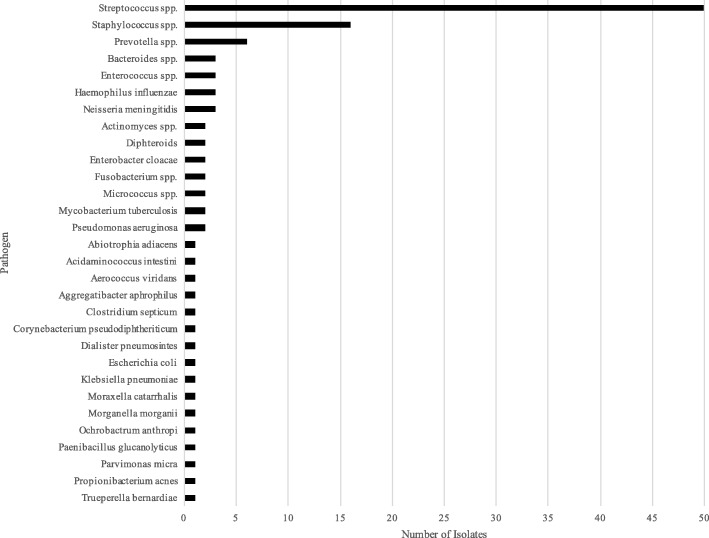
Distribution of Isolated Pathogens in Growth-Positive Cultures (*N* = 114)

60/93 patients (74.2%) had blood cultures taken before antibiotic treatment and 17 were positive (28.3%). CSF cultures were taken in 38/93 patients (40.8%) and 18 were positive (47.4%). Intracranial pus was cultured in 70/93 patients (75.3%) and positive for 52 (74.3%). Paranasal sinus cultures were taken from 23/93 patients (24.7%) and positive in 14 (60.9%). Overall 114 isolates of 53 species were grown amongst 69 patients (Fig. 4). 50/114 isolates were *Streptococcus spp.*, of which *Streptococcus milleri* group organisms and *Streptococcus pneumoniae* were most often isolated, 25 and 8 times respectively. *Streptococcus pyogenes* was isolated 5 times.

16/114 isolates were *Staphyloccus spp.*, of which 9 *Staphyloccus aureus*, and 7 coagulase-negative *Staphylococci*. Coagulase-negative *Staphylococci* were only considered relevant if isolated from intracranial pus culture.

6 culture-negative patients had PCR-positive samples for either *Streptococcus intermedius, Streptococcus pneumoniae, Fusobacterium nucleatum, Neisseria meningitidis* or *Aspergillus spp.* Additionally, 4 patients had microscopically positive samples for gram-positive cocci. In 13 patients all microbiological investigations were negative.

In 21 patients (22.6%) multiple microorganisms were isolated from different sites (range, 2–6 microorganisms) and 12 patients (12.9%) had polymicrobial abscess or empyema (range, 2–6 microorganisms). 21 anaerobic species were isolated from 12 patients, most commonly Prevotella spp. Anaerobic microorganisms were only isolated from children aged 1–15 years.

### Antibiotic treatment

92 patients were started on antibiotics and 3 on antifungals. 90 patients were started on empirical antibiotic regimens and two on quadruple therapy for tuberculosis.

Empirical antibiotic regimens consisted of 1–4 antibiotics and all but tuberculostatics were initially administered intravenously. 17 different antibiotics were given in 31 empirical regimens.

Most commonly administered empirical regimens consisted of a third-generation cephalosporin, metronidazole and amoxicillin in 29/90 patients (32.2%). 7 patients had a third-generation cephalosporin alone, 15 had a third-generation cephalosporin plus metronidazole, and 13 a third-generation cephalosporin plus metronidazole and a third antibiotic other than amoxicillin.

Antibiotic changes occurred in 86 patients (95.6%). Reasons documented included microbiology results, antibiotic sensitivities, adverse reactions, and conversion to oral administration. 13 patients (14.4%) developed antibiotic-related rashes and 6 antibiotic-related neutropenia. 18 patients required additional teicoplanin for presumed central line infections.

61 patients were converted to oral antibiotics (66.3%) and 5 children received intrathecal antibiotics. Most oral regimens consisted of amoxicillin, amoxicillin/clavulanic acid, and/or metronidazole. Overall, 22 different antibiotics were used in this cohort.

Total duration of antibiotic treatment was documented for 89 patients, intravenous duration for 90 and oral treatment for 61. For 3 patients, antibiotic treatment duration was unclear; 1 had an unclear end of intravenous treatment, and 2 an unclear end of oral treatment.

6 patients were excluded from antibiotic treatment duration analysis; 2 had *Mycobacterium turberculosis* and 2 had *Actinomyces spp*. infection, both known to require prolonged antibiotic treatment, one had complex *Clostridium septicum* infection requiring individualised prolonged antibiotic treatment and one patient died 9 days into treatment, not completing the intended antibiotic course.

Median total duration of antibiotic treatment was 92 days (IQR, 59–119 days), median duration of intravenous antibiotics 46.50 days (IQR, 25–71.50 days), and median duration of oral antibiotics 47 days (IQR, 38–77 days).

### Antibiotic coverage

Sixty-nine patients on empirical antibiotics had growth-positive cultures. For 63/69 patients full sensitivities were available and antibiotic coverage was analysed.

Chosen empirical regimens provided sufficient coverage in 60/63 patients (95.2%). 2 patients had resistant microorganisms; one a metronidazole-resistant *Dialister pneumosintes*, and one an amoxicillin and cefotaxime-resistant *Ochrobactrum anthropi*. 31/63 patients (49.2%) had an empirical regimen containing a third-generation cephalosporin or carbapenem plus amoxicillin, of which 30 did not require amoxicillin for additional coverage. One patient for whom amoxicillin provided necessary coverage grew a cefotaxime-resistant, amoxicillin-sensitive *Enterococcus faecalis.* The combination of a third-generation cephalosporin plus metronidazole would have been sufficient in 57/63 patients (90.5%), and a third-generation cephalosporin alone in 51/63 (81.0%). Meropenem plus linezolid would have covered 60/63 patients (95.2%). Linezolid was analysed rather than vancomycin because of superior CNS penetration which would be preferred despite potential side effects of linezolid (bone marrow suppression and neuropathy), as these are usually not observed in the first four weeks of treatment [25].

### Neurosurgery

75/93 patients required neurosurgery (80.6%). 35 patients underwent craniotomy, 30 burr hole drainage, 4 craniectomy, 4 aspiration via fontanelle, and 2 stereotactic aspiration. Median duration to neurosurgery was one day from admission (IQR 0–3 days). 30 patients required repeated neurosurgical interventions (maximum 6).

### Outcome

Mortality in this cohort was 3.2%. Median duration of hospitalisation was 23 days (IQR, 15.5–34.5 days). Children exclusively treated with intravenous antibiotics did not require longer hospitalisation than children converted to oral antibiotics (median 25 versus 22 days, *p* = 0.266).

Follow-up data was available for 87/92 surviving patients (93.4%). One patient was lost to follow-up due to emigration and 4 had an unclear follow-up duration. 10 patients were still in follow-up. For patients with completed follow-up the median duration was 7.99 months (IQR 4.92–12.64 months). Recurrence of intracranial suppurative infection occurred once in a child who developed bone flap osteomyelitis post-antibiotic treatment.

Thirty-five children (38.0%) had short-term neurological sequelae (< 6 months post-discharge) not present at initial presentation and 22 children (23.9%) long-term neurological sequelae (≥6 months post-discharge). Most common were seizures and hemiplegia (Table 3). Fifteen patients resolved short-term neurological sequelae within 6 months and 2 developed neurological sequelae ≥6 months post-discharge.

**Table 3:** Neurological Sequelae < 6 months and ≥ 6 months post-discharge not present at admission

Neurological sequelae	< 6 months post-discharge (n = 92)	≥6 months post-discharge (*n* = 92)	Neurological sequelae	< 6 months post-discharge (n = 92)	≥6 months post-discharge (n = 92)
Any sequelae	35 (38.0%)	22 (23.9%)	Any sequelae	35 (38.0%)	22 (23.9%)
Seizures	10 (28.6%)	11 (50%)	Nystagmus	2 (5.7%)	2 (9.1%)
Hemiplegia	6 (17.1%)	4 (18.1%)	Torticollis	2 (5.7%)	–
Diplopia	4 (11.4%)	3 (13.6%)	Dysdiadochokinesis	1 (2.9%)	–
n.VII palsy	4 (11.4%)	3 (13.6%)	Hearing loss	1 (2.9%)	2 (9.1%)
Speech abnormality	4 (11.4%)	1 (4.5%)	Hemianopia	1 (2.9%)	2 (9.1%)
Gait abnormality	3 (8.6%)	3 (13.6%)	Neurodevelopmental delay	1 (2.9%)	1 (4.5%)
Dysphasia	3 (8.6%)	3 (13.6%)	Memory difficulties	1 (2.9%)	2 (9.1%)
Delayed motor development	2 (5.7%)	–	Visual discrimination difficulty	1 (2.9%)	–
Hemiparesis	2 (5.7%)	2 (9.1%)	Learning difficulties	–	2 (9.1%)
Hydrocephalus	2 (5.7%)	3 (13.6%)	Concentration difficulties	–	1 (4.5%)
n. VI palsy	2 (5.7%)	–	Cerebral palsy	–	1 (4.5%)
Neuropathic pain	2 (5.7%)	1 (4.5%)			

Median symptom duration before admission was shorter for children with short-term neurological sequelae (6 versus 10 days, *p* = 0.002) and with long-term neurological sequelae (5 versus 8.50 days, *p* = 0.037), compared to patients without neurological sequelae. Initial Glascow Coma Scale (GCS) was not recorded for all patients, prohibiting meaningful statistical analysis. Although there seemed to be a trend for patients with a low GCS to have worse outcomes, also patients with maximal GCS, but who deteriorated rapidly after admission tended to have worse outcomes.

Conversion to oral antibiotics was not associated with development of short- (*p* = 0.959) or long-term (*p* = 0.135) neurological sequelae, compared to children exclusively on intravenous antibiotics.

Patients with unusual clinical courses or where *S. pneumoniae* was cultured were investigated for immunodeficiencies. Only one patient with underlying immunodeficiency was identified.

## Discussion

Our study is the largest single-centre study assessing local management of paediatric focal intracranial suppurative infection in order to analyse effectiveness of local management, identify causative pathogens and guide empirical antibiotic therapy. Our findings extended on Cole et al. [9] and demonstrated similarities and differences compared to case-series by other centres.

Our estimated annual incidence was higher than the 5.3/1,000,000 previously reported in the UK. [12] The annual incidence in this tertiary centre was three times higher than in other developed countries. [3, 11, 16, 18, 20, 21] Our local annual incidence showed similarity with a Cameroonian study. [17] Reasons for this may lie in high rates for non-specific symptoms and subsequent lower suspicion for focal intracranial suppuration, and less health-seeking behaviour for upper respiratory tract infections. Median age at admission and bimodal age distribution are consistent with other studies. [12, 17]

Mortality in this cohort was 3.2%, which is at the lower end of the spectrum previously reported (2.6–21.4%). [3, 7, 11, 12, 15–18, 20–22] Neurological morbidity remains a significant problem, yet rates concur with other centres. [11, 12, 17, 18, 31] Long-term neurological sequelae affected a smaller proportion than short-term neurological sequelae, but might be underreported as follow-up in tertiary care ended, and neuropsychological effects and mild cognitive impairments are difficult to diagnose.

Predisposing factors were identified in 90.5%. Meningitis was associated with younger and sinusitis with older age, which is unsurprising given sinus maturation. High rates of sinusitis and meningitis may be explained by the number of SDE patients in this cohort. There is an association between SDE and meningitis and sinusitis, previously demonstrated by Legrand et al. [16] Adolescent patients are more at risk for intracranial complications of sinusitis compared to younger children and adults, and sinusitis patients were mostly adolescents. [32] Adolescents are more likely to develop intracranial extension of sinusitis, as vascularity and blood supply of diploic veins is known to be increased compared to adults. [33] CHD as a predisposing factor in this study was rare. This has been reported by other centres [3, 17, 31], although some centres report CHD as their main predisposing factor in 20.8–40%. [7, 18, 20, 21]

35.6% had focal neurological deficits at presentation and 23.7% seizures at admission. This is similar to other centres [11, 17, 18], although rates go up to 53% [12] and 48% [21] respectively. In this study the most common symptoms were non-specific. The classic triad was seldom seen. This is in accordance to literature from developed countries [3, 18], while developing countries see the triad in up to 52% [17]. Children with poorer outcome had shorter symptom duration, and tended to have more severe altered levels of consciousness, which may be attributed to more rapid deterioration. Although otorhinolaryngological infections were prevalent in this cohort, concurrent symptoms were not often reported and may be underreported since patients often had more severe presenting clinical features.

*Streptococci* were most commonly isolated with *Streptococcus milleri* group organisms as most common species. The second largest group were *Staphylococci*, although it is debatable how many coagulase-negative isolates were pathogenic. The predominance of *Streptococci* has been described previously in the UK [12], as well as in other countries [3, 7, 11, 16, 20–22, 31]. 12.9% of isolates were anaerobic which concurs with published literature. [11, 12] Interestingly *Prevotella spp*. were most common, whereas other centres report *Fusobacterium spp*. as the most common anaerobic isolate. [3, 11, 20]

This study showed great variability in empirical regimens and in antibiotic treatment duration. This has been noted previously [3, 11, 12], and demonstrates the lack of uniform guidelines and potentially a change in practice over the 15 years covered by the study. Nonetheless, chosen regimens provided sufficient coverage in the majority of patients. Broad-spectrum antibiotics remain necessary before microbiology results, as gram-positive, gram-negative and anaerobe microorganisms were all cultured in this cohort. Combinations including third-generation cephalosporins plus metronidazole were successful in the majority of patients. 90.5% of patients with growth-positive cultures would have been sufficiently covered with a third-generation cephalosporin plus metronidazole, and most patients with negative cultures improved clinically on this regime. Therefore, we recommend this regime as first-choice empirical antibiotic treatment. Despite meropenem plus linezolid covering a greater proportion, this regimen should be reserved for severe or treatment non-responsive cases. Over the 15.5-year period, there have not been any anaerobic pathogens cultured in the < 1 and > 15-years age groups. In infants, the routine use of metronidazole might be reconsidered if anaerobic cultures are negative. The > 15-years age group was too small to make a similar recommendation.

### Strengths and limitations

This study has the largest number of paediatric patients in a single-centre cohort. This enabled us to comprehensively review the management of paediatric focal intracranial suppurative infections.

Limitations lie in the retrospective nature of the study, leading to potential information bias. 2.8% of cases had to be excluded because of unavailable case notes. Long-term complications possibly have been underreported as follow-up ended and patients may have presented with sequelae to local hospitals or have subtler, underrecognised, neuropsychological sequelae. The single-centre aspect of this study makes results difficult to generalise, and there are differences compared to other single-centre studies. However, it shares similarities with other centres and provides additional data to assist clinical choices in centres with similar microbiological profiles and facilities.

In conclusion, paediatric focal intracranial suppurative infection continues to cause significant mortality and morbidity. Although uncommon, it occurs more frequently in the North East of England than previously reported in developed countries. Empirical antibiotic regimens should provide broad-spectrum coverage and we recommend a third-generation cephalosporin plus metronidazole. Meropenem and linezolid should be reserved for severe and complex cases. In infants the use of metronidazole might be reconsidered if the microbiology results are negative for anaerobes. Optimum duration of antibiotic treatment remains unclear. A multi-centred, prospective randomised controlled trial would be required to answer this question. Further research should focus on the identification of factors causing the local higher occurrence paediatric focal intracranial suppurative infection and the development of guidelines for antibiotic treatment.

## Abbreviations

BA: brain abscess
CGD: chronic granulomatous disease
CHD: congenital heart disease
CRP: C-reactive protein
CSF: Cerebrospinal fluid
EDE: extradural empyema
GCS: Glasgow Coma Scale
GNCH: Great North Children’s Hospital
IQR: interquartile range
PCR: polymerase chain-reaction
PICU: Paediatric Intensive Care Unit
SDE: subdural empyema
UK: United Kingdom
